# Doxazosin and Carvedilol Treatment Improves Hepatic Regeneration in a Hamster Model of Cirrhosis

**DOI:** 10.1155/2018/4706976

**Published:** 2018-12-12

**Authors:** Sandra Alejandra Serna-Salas, Yesenia Danyeli Navarro-González, Sandra Luz Martínez-Hernández, Luis Fernando Barba-Gallardo, Esperanza Sánchez-Alemán, Liseth Rubí Aldaba-Muruato, José Roberto Macías-Pérez, Javier Ventura-Juárez, Martin Humberto Muñoz-Ortega

**Affiliations:** ^1^Morphology Department, Basic Sciences Center, Autonomous University of Aguascalientes, Mexico; ^2^Pharmacology and Physiology Department, Basic Sciences Center, Autonomous University of Aguascalientes, Mexico; ^3^Microbiology Department, Basic Sciences Center, Autonomous University of Aguascalientes, Mexico; ^4^Optometry Department, Center for Health Sciences, Autonomous University of Aguascalientes, Mexico; ^5^Clinical Chemistry, Autonomous University of San Luis Potosí, Multidisciplinary Academic Unit, Huasteca Zone, Mexico; ^6^Chemistry Department, Basic Sciences Center, Autonomous University of Aguascalientes, Mexico

## Abstract

Regulation of the mechanisms of fibrosis is an important goal in the treatment of liver cirrhosis. One mechanism is the participation of hepatic stellate cells in fibrogenesis when activated by catecholamines. Consequently, *α*/*β* adrenoblockers are proposed as an alternative treatment for chronic liver lesions such as fibrosis and/or cirrhosis and for possible liver regeneration. We herein analyzed the effect of doxazosin and carvedilol treatments during the regeneration of tissue in a hamster model of liver cirrhosis. Tissue samples were examined by H&E and PAS to evaluate tissue damage and with Sirius red to assess collagen fiber content. ALT, AST, albumin, and total proteins were examined by spectrophotometry. Determination of the levels of *α*-SMA and TGF-*β* in hepatic tissue was examined by Western blot and of the expression of TIMP-2, MMP-13, *α*-FP, HGF, CK-7, and c-Myc was examined by qPCR. Treatment with doxazosin or carvedilol prompted histological recovery and reduced collagen fibers in the livers of cirrhotic hamsters. The expression of TIMP-2 decreased and that of MMP-13 increases in animals treated with adrenoblockers with respect to the group with cirrhosis. Additionally, the concentration of *α*-SMA and TGF-*β* declined with both drugs with respect to placebo p<0.05. On the other hand, each drug treatment led to a distinct scenario for cell proliferation markers. Whereas doxazosin produced no irregularities in *α*-FP, Ki-67, and c-Myc expression, carvedilol induced an increment in the expression of these markers with respect to the intact. Hence, doxazosin and carvedilol are potential treatments for the regression of hepatic cirrhosis in hamsters in relation to the decrease of collagen in the hepatic parenchyma. However, at regeneration level we observed that doxazosin caused slight morphological changes in hepatocytes, such as its balonization without affecting the hepatic function, and on the other hand, carvedilol elicited a slight irregular expression of cell proliferation markers.

## 1. Introduction

Cirrhosis is a syndrome of chronic liver damage caused by multiple etiologies. It is characterized by the loss of liver parenchyma as well as the formation of fibrous septa and structurally abnormal regenerative nodules, resulting in a distortion of the normal hepatic architecture. These histopathological alterations affect the vascular physiology of the portal system [1]. The biochemical profile for this syndrome includes an elevation in the secretion of ALT, AST, GGT, and decreased synthesis of albumin, total proteins, and glycogen [1–3] and* in situ* increased deposit of alpha-1 type I collagen as well as alterations in the levels of transforming growth factor *β* (TGF-*β*), tissue inhibitor of metalloproteinases 1 (TIMP-1), tissue inhibitor of metalloproteinases 2 (TIMP-2), metalloproteinase 1,2,4,5 (MMP), and platelet-derived growth factor (PDGF). These molecules are synthesized by activated hepatic stellate cells (HSCs) Alpha Smooth Muscle Actin (*α*-SMA) positives, which are mainly responsible for the development of the fibrogenic process during liver cirrhosis [2–8]. Hence, HSCs are a therapeutic target for the reversion of this disorder.

On the other hand, the use of *α*/*β*-adrenergic receptors antagonists has been the treatment of choice to prevent hepatic hypertensive diseases [9–11]. Carvedilol (an *α*/*β* adrenoblocker) is used as an antihypertensive drug. It is used clinically to reduce portal hypertension in patients with liver damage and exerts antioxidant and antifibrotic effects [11–13]. The doxazosin (*α*1-adrenoblocker) has hypotensive activity in arteries through the inhibition of alpha 1 adrenoblocker. In addition, doxazosin is used for the treatment of benign prostatic hyperplasia and arterial hypertension. Doxazosin elicits a decrease in fibrosis by modulating the activity of renal mesangial cells and collagen-producing myocardial fibroblasts by an as yet unknown mechanism [14–16]. During the development of liver cirrhosis in hamsters, the latter drug is also able to improve liver function by reversing fibrosis generation [17, 18].

HSCs express *α*/*β*-adrenergic receptors, which trigger the production of type I collagen when stimulated with noradrenaline. Furthermore, noradrenaline was reported to induce the proliferation of stellate cells through several signaling pathways, such as p38, MAP, PI3K, PKC, and MEK. The same study demonstrated that *α*/*β* adrenoblockers decrease the activation and proliferation of HSCs [19, 20].

Cirrhosis of the liver leads to hepatocytes death as an effect of continued exposure to a harmful agent. According to clinical findings, acute damage due to chemical poisoning, viruses, metabolic abnormalities, and vascular disorders causes a loss of liver tissue that compromises the ability of the organ to perform its vital functions. These events are associated with inflammation as well as a regenerative response [21, 22]. Indeed, the liver has a great capacity for regeneration, possibly because of its robust population of progenitor and neuroendocrine cells [23, 24].

The regenerative response of hepatocytes is initiated by endothelial cell proliferation, creating small vascular spaces in hepatic sinusoids [24]. Endothelial cells are stimulated by angiogenic factors produced by hepatocytes, including vascular endothelial growth factor (VEGF), fibroblast growth factor (FGF), and TGF-*α*. On the other hand, hepatocytes are triggered by the epithelial growth factor (EGF) and hepatic growth factor (HGF) to synthesize DNA and therefore augment liver tissue [24–26], this in face of an ideal situation of regeneration. On the other hand, it is also known that, during chronic damage, the liver expresses an increase of embryonic proteins such as alpha-fetoprotein (*α*-FP), as well as the expression of the oncogene c-MyC; these are markers for hepatic proliferation, although a sustained increase in these markers predicts a possible neoplastic process [27]. As has been observed in mouse and rat models, there is a periportal population of small primitive cells of epithelial origin that promote the multiplication of hepatocytes. These cells, denominated oval cells or hepatic progenitor cells (HPC), are related to the bile ducts even during their differentiation [28, 29]. In the initial stages of differentiation, they are called hepatobiliary cells [21, 24, 26, 30]. They display markers for immature hepatocytes (e.g., *α*-fetoprotein (*α*-FP)) and for biliary cells (e.g., cytokeratins- (CK-) 7 and CK-19) [30]. According to the current results, the administration of *α*/*β*-adrenoblockers after a chronic injury may foster the reversal of liver cirrhosis through the promotion of cell regeneration, thus contributing to the improvement of liver function. Hence, the purpose of the present study was to evaluate the effect of doxazosin and carvedilol at the level of biochemical markers of liver damage and regenerative processes in a hamster model of CCl4-induced liver cirrhosis.

## 2. Materials and Methods

### 2.1. Induction of Liver Cirrhosis in Hamsters

**Figure 1:**
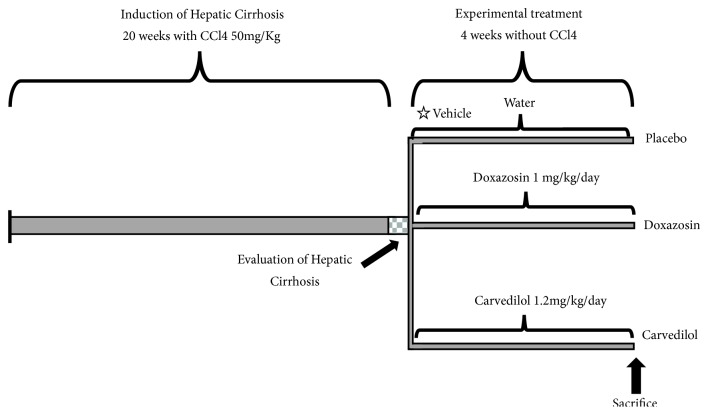
Experimental design timeline, where times and treatments for the control and experimental groups are represented: cirrhosis, placebo, and treated with alpha and beta adrenoblockers.

Twenty-five 6- to 8-week-old male golden hamsters (*Mesocricetus auratus*, 100-150 g) were maintained on a light/dark cycle (12:12) and provided Purina Rodent Chow and water* ad libitum*. The experiment began with two groups: (i) the intact (control) group (n=5) and (ii) the cirrhosis group (n=20). Cirrhosis was induced by the intraperitoneal administration of 50 mg/kg CCl4 in petrolatum, 2 times per week (n=20) for 20 weeks [17, 18]. Each animal was weighed once per week throughout the experiment. To determine the degree of fibrosis/cirrhosis by morphometry and qPCR, the 5 control hamsters and 5 of the animals from the cirrhosis group were sacrificed (Figure 1). Photographs of livers were taken* in situ* of the anterior and visceral faces with an Olympus xD Master 2 model SP-55OUZ camera. Subsequently, hamsters were sacrificed with a pentobarbital overdose (MAVER Laboratories). All animal experiments were approved by the Animal Welfare and Research Ethics Committee of the Autonomous University of Aguascalientes, and were conducted in accordance with institutional and national regulations (NOM-062-ZOO-1999). Serum samples were taken to quantify the levels of alanine aminotransferase (ALT) and aspartate transaminase (AST), and pieces of tissue were fixed in neutral formalin at 4% for the histological analysis of liver damage. In addition, liver tissue samples were taken to establish the qPCR expression of TIMP-2, metalloproteinase 13 (MMP-13), *α*-FP, HGF, CK-7, and the oncogene c-Myc.

### 2.2. Application of *α*/*β*-Adrenergic Blockers

The remaining 15 hamsters with cirrhosis were divided into three groups (n=5 each) with the following 4-week treatments: (1) carvedilol (1.2 mg/Kg/day, (2) doxazosin (1 mg/Kg/day, and (3) placebo (control of endogenous reversal) (Figure 1); the last one is important to check the activity of the adrenoblockers on hepatic cirrhosis and not only the efficiency of the animal model to reverse the damage on its own. The doses and frequency of administration were based on previous studies [17, 18]. Subsequently, the hamsters were sacrificed and the liver samples were fixed in neutral formalin at 4% for histological examination. Later, RNA was isolated for qPCR. Serum samples were also obtained to measure the level of liver function markers.

### 2.3. Histological Technique

Hepatic tissue samples fixed in 4% formalin were processed by the paraffin technique to obtain 5-micron thick sections, cut on a rotating microtome (Leica RM 2125RT). In histological sections, the following staining was performed: (i) hematoxylin/eosin (H&E) for analysis of the histopathology of treated animals, (ii) PAS for the evaluation of glycogen synthesis and storage capacity, and (iii) Syrian red for the determination of collagen fibers deposits (type I = red, type III = green) by polarized light microscopy. The histological preparations were visualized using a Zeiss Axioscope 40/40 FL microscope and analyzed with the Image Pro Plus Software 4.5.1. For the quantification of the fibrotic area, a previously reported methodology was employed [17]. Briefly, color images were converted to 8-bit grayscale format in Adobe Photoshop CS5 (Adobe Systems, San Jose, CA) after composite the figures. This procedure was applied equally across images within a data set. The intensity of each zone was measured to determine mean intensity per square micrometer in Fiji (ImageJ distribution) software [31], and then, it was compared with the total area.

### 2.4. Liver Function Markers

AST, ALT, albumin, and total proteins were measured by spectrophotometry (Biosystems bts-350) in serum obtained from blood extracted by cardiac puncture.

### 2.5. Protein Extraction and Western Blot Detection of *α*-SMA and TGF-*β* in Hepatocytes

To evaluate the presence of alpha smooth muscle actin (*α*-SMA) and TGF-*β* in the liver parenchyma, 100 *μ*g of tissue was homogenized in 1 mL of lysis buffer (50 mM Tris-HCl at pH 6.8, 5 mM N-ethylmaleimide, 3 mM iodoacetamide, 1 mM phenylmethanesulfonyl fluoride, and 3 mM tosyl-L-lysine chloromethyl ketone) for total protein extraction. The lysate was centrifuged at 40,000 g for 1 h at 4°C. The supernatants were suspended with 200 *μ*L lysis buffer and 1% triton X-100. Protein quantification was performed by the Bradford method.

For Western blot, 50 *μ*g of each protein extract was separated in a 12% SDS-PAGE gel and proteins were transferred to a PVDF membrane (Bio-Rad, 162-0176, Hercules, USA). Blockage was carried out for 1 h at room temperature with TBST (Tris-buffered saline/0.05% Tween-20) and 5% skimmed milk. For immunodetection, the membrane was incubated for 1 h at room temperature with the following antibodies diluted at 1:1000: mouse anti-human TGF-*β* (Peprotech H2614), rabbit polyclonal anti-*α*-SMA (Abcam ab5694), and rabbit polyclonal anti *β*-actin (Abcam ab69512). Then, blots were incubated with the conjugated antibody, goat anti-mouse, and goat anti-rabbit marked with alkaline phosphatase 1:2000 (Sigma A3688, Abcam ab6722). After three washes with TBS, blotting was developed by alkaline phosphatase with the sigma fast bcip/nbt (Sigma).

### 2.6. Isolation of Total RNA and RT-PCR

Total RNA was isolated from 100 mg of liver tissue of the control and experimental animals with the SV Total RNA Isolation System (Promega, Madison, WI, USA), according to the manufacturer's protocol. Total RNA was quantified with a NanoDrop-2000 (Thermo Scientific, Waltham, MA, USA) and stored at −80°C until needed. Reverse transcription was performed with 1 *μ*g of total RNA with the GoScript Reverse Transcription System (Promega). Subsequently, real-time quantitative PCR was performed by using the qPCR green Master with UNG-clear (Jena Bioscience, Jena, Germany) on a StepOne apparatus (Applied Biosystems) in the following manner: 50°C for 2 min, 95°C for 45 sec, 40 cycles of 95°C for 45 sec, and 60°C for 45 sec. Oligonucleotides were designed to target MMP-13, TIMP-2, HGF, c-MYC, *α*-FP, and *β*-actin as reference controls (Table 1). Relative expression levels were normalized against *β*-actin as an internal housekeeping gene and differences were determined by employing the ΔΔCt relative method.

### 2.7. Statistical Analysis

GraphPad Prism V5 software was employed for statistical analysis and figures. Data were expressed as the mean ± standard error of the mean (SEM) of each group. Significant differences between mean values were assessed by using the two-way analysis of variance test with the Tukey post hoc. Statistical significance was considered at p<0.05.

### 2.8. Immunohistochemical Analysis for Ki-67

Immunohistochemistry was performed to analyze Ki-67 positive cells in liver tissues. Briefly, liver tissue slides were incubated with a rabbit polyclonal anti-Ki-67 (Abcam, 15580) for 12 h at 4°C. As a secondary antibody, we used an Envision + Dual Link System-HRP (Dako, California, USA). The peroxidase activity was developed with Sigma Fast Diaminobenzidine (Sigma-Aldrich); the number of Ki-67-positive cells was counted in the entire histological section and reported as cells/mm^2^. Data was documented in a Zeiss Axioscope 40/40FL microscope (Zeiss, Oberkochen, GE) and analyzed with the Image Pro Plus Software 4.5.1 (Media Cybernetics, Bethesda, MD, USA).

## 3. Results

### 3.1. *α*/*β* Adrenoblockers Improved Liver Histology in Hamsters after CCl4-Induced Cirrhosis

**Figure 2:**
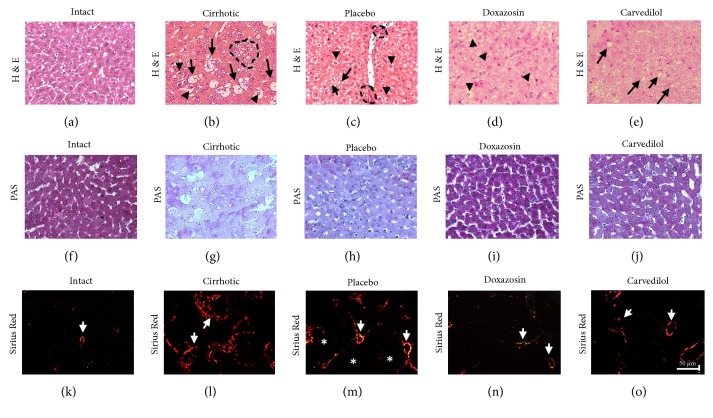
Analysis of the histological structure of the hepatic parenchyma after treatment with *α*/*β* adrenoblockers, carried out with H&E, PAS and Sirius red. (a), (f), and (k) Intact animals. (b), (g), and (l) Group with cirrhosis. (c), (h), and (m) Placebo group. (d), (i), and (n) Group treatment with doxazosin. (e), (j), and (o) Group treatment with carvedilol. White arrow, indicated fibrotic area, dotted line indicated inflammatory infiltrate and cellular damage, black arrow indicated steatosis, black arrowhead indicated swollen spherical hepatocytes and cellular edema, and asterisks indicated regeneration nodules.

In the cirrhosis group (sacrificed without treatment), there was steatosis (Figure 2(b), black arrow), necrosis and hepatocellular degeneration (Figure 2(b), dotted line), as well as the formation of fibrous tissue type I (Figure 2(l), white arrow), the development of areas with inflammatory cell infiltration, and a decrease in glycogen content (Figure 2(g)). Compared to the cirrhotic animals, the placebo group exhibited scarce areas of inflammatory infiltrate (Figure 2(c), dotted line) and cellular edema (Figure 2(c), arrowhead), a diminished glycogen content (Figure 2(h)), an important area of collagen fibers type I (Figure 2(m), white arrow), and a larger area of regenerative nodules (Figure 2(m), asterisk) indicating that the hamster could reverse liver damage endogenously. The livers of animals treated with carvedilol or doxazosin showed a marked decrease in fibrous tissue type I (Figures 2(n) and 2(o), white arrows) and the normalization of liver histology compared to the cirrhosis and placebo groups (Figures 2(l) and 2(m)); with both drug treatments, liver tissue displayed characteristics similar to those in the intact group (Figures 2(a), 2(f), and 2(k)). The doxazosin treatment led to a significant partial rearrangement of the hepatic structure and restoration of glycogen synthesis (Figure 2(i)). Swollen spherical hepatocytes were detected at the cellular level (Figure 2(d), arrowhead).

### 3.2. The Application of *α*/*β* Adrenoblockers (Doxazosin and Carvedilol) Improved Liver Function and Reduced the Amount of Fibrotic Tissue

**Figure 3:**
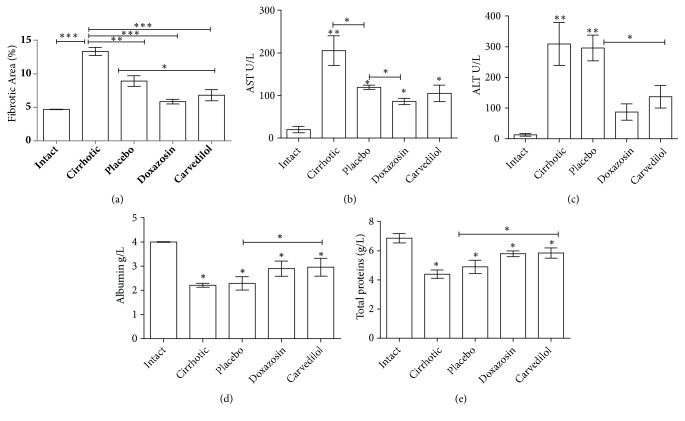
Effect of the reduction of fibrosis and recovery of the hepatic function through the administration of the *α*/*β* adrenoblockers, doxazosin and carvedilol. Compared to the animals with cirrhosis and without treatment, those treated with either of the test drugs showed a clear reduction in the percentage of hepatic fibrosis (a), a decrease in the enzymes related to cellular damage (e.g., ALT and AST), and an increase in the biosynthetic capacity of albumin and total proteins (b-e). It was evaluated with analysis of variance test with the Tukey post hoc values which are expressed as the mean ± SD. ^*∗*^p < 0.05, ^*∗∗*^p < 0.01, and ^*∗∗∗*^p < 0.001 for the treated groups versus the intact and cirrhosis groups (n=5).

Compared to intact animals, an increase in collagen deposits was found in the cirrhosis (p<0.001) and placebo groups (p<0.05) (Figures 2(l), 2(m), and 3(a)). Compared to the latter two groups, there was a significant drop in type 1 collagen per unit area with the doxazosin and carvedilol treatments, resulting in values similar to the intact group (Figures 2(n), 2(o), and 3(a)).

Compared to the intact animals, the cirrhosis group had significantly higher levels of ALT (~220 fold) and AST (~300 fold) (p<0.001). Regarding albumin and total proteins, a marked decrease in the biosynthetic capacity of the hepatic parenchyma was observed in the cirrhosis and placebo groups (p<0.05). Compared to these latter two groups, the hamsters treated with doxazosin showed lower values of both transaminases, while the animals treated with carvedilol only exhibited a reduction in the ALT enzyme. In all these cases, the respective values were similar to those observed in the intact group (Figures 3(b) and 3(c)).

### 3.3. *α*/*β* Adrenoblockers (Doxazosin and Carvedilol) Diminished *α*-SMA and TGF-*β* Levels in the Cirrhotic Hamsters

**Figure 4:**
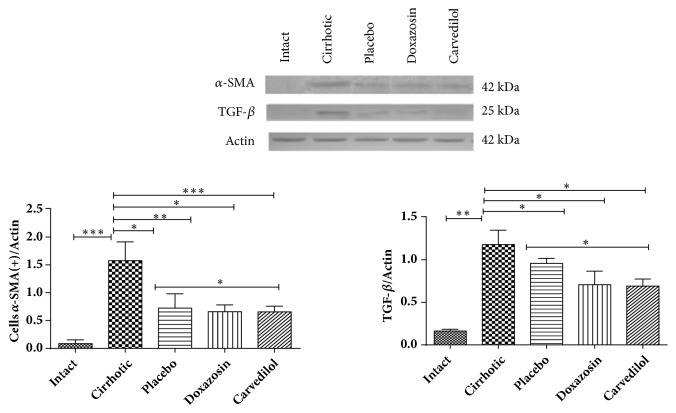
Expression and quantification of *α*-SMA for activated hepatic stellate cells (HSCs) and TGF-*β*, demonstrating the antifibrotic capacity of the treatments with doxazosin and carvedilol. It was evaluated with analysis of variance test with the Tukey post hoc. Values are expressed as the mean ± SD for each of the markers of cirrhosis currently analyzed (n=3 tissue samples for each group). ^*∗*^p < 0.05 doxazosin and carvedilol versus placebo for *α*-SMA and TGF-*β* and ^*∗∗∗*^p < 0.001. Intact versus cirrhotic for *α*-SMA and TGF-*β*.

To evaluate the possible antifibrotic effect of the two adrenoblockers herein tested, analysis was made of HSCs in their active form by identifying *α*-SMA and TGF-*β* (the latter because of its profibrogenic function). By Western blot of liver tissue, a 7.5-fold increase was detected in the *α*-SMA protein in the cirrhotic versus intact animals (p<0.001) (Figure 4), which suggests the presence of HSCs in their active state in the former group. On the other hand, the adrenoblockers achieved at least a 2.5-fold decrease in the concentration of *α*-SMA with respect to the cirrhosis group (p<0.05). The placebo group had a similar reduction in the concentration of *α*-SMA as the animals treated with doxazosin and carvedilol (p<0.05), perhaps due to remodeling in the hepatic parenchyma fomented by the larger regenerative nodules. The TGF-*β* protein was examined as an indicator of fibrotic activity. The level of this protein was at least 4-fold higher in the cirrhosis versus intact and placebo groups (p<0.01). In contrast, treatment with the adrenoblockers caused a significant drop the production of TGF-*β* compared to the cirrhosis and placebo groups (p<0.05). The levels of both proteins (*α*-SMA and TGF-*β*) decreased appreciably in animals administered with doxazosin and carvedilol, even though basal values were not reached. The decline found in the concentration of these proteins in relation to the placebo group provides evidence of the effectiveness of the drugs attenuating the process of fibrogenesis promoted by activated HSCs (Figure 4).

### 3.4. *α*/*β* Adrenoblockers (Doxazosin and Carvedilol) Improved the Process of Liver Cirrhosis Reversal and Cell Regeneration

**Figure 5:**
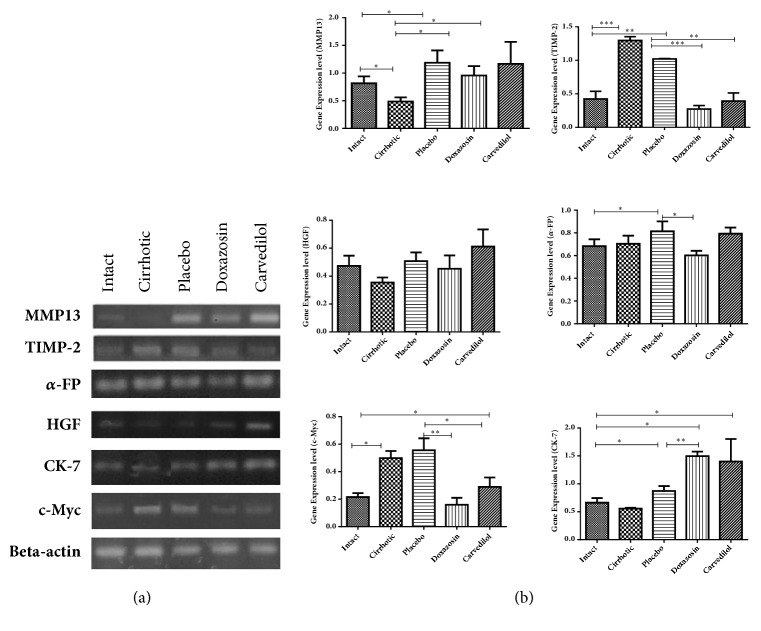
Expression of the markers of regulation of extracellular matrix and proliferation or carcinogenesis, showing the degree of regeneration. (a) The electrophoresis gel shows the different markers evaluated by primers in endpoint PCR (MMP-13, TIMP-2, *α*-FP, HGF, CK-7, and c-Myc). (b) Quantitative analysis by qPCR shows the level relative expression (axis “Y”) for each marker (MMP-13, TIMP-2, *α*-FP, HGF, CK-7, and c-Myc). It was evaluated with analysis of variance test with the Tukey post hoc. The bars represent the mean value ± SD. ^*∗*^p < 0.05; ^*∗∗*^p < 0.01; ^*∗∗∗*^p < 0.001 of the treated groups in relation to the intact and cirrhosis (without treatment) groups (n=5).

To verify that doxazosin and carvedilol have the capacity to enhance cell regeneration and also to reverse histological damage, the expression of different markers of cell division and fibrosis was analyzed by using qPCR. After 20 weeks of treatment with CCl4, the hamsters of the cirrhosis group displayed alterations in the expression of genes responsible for regulating the degradation of the extracellular matrix, such as MMP-13 and TIMP-2. Compared to the intact group, the group with cirrhosis exhibited a decrease in the level of MMP-13 and a 3-fold increase in the level of TIMP-2 (Figure 5(b)). On the other hand, compared to the cirrhosis and placebo groups, the animals treated with doxazosin and carvedilol showed a 2-fold greater expression of MMP-13 and a lower expression of TIMP-2 (p<0.05), both of which reached levels similar to the intact group. The treatments with doxazosin and carvedilol demonstrated an adequate relation between MMP and TIMPs, as evidenced by the elevated expression of the MMP-13 and the reduced expression of TIMP-2. This confirms that both drugs regulate the process of degradation of accumulated extracellular matrix.

To test whether the process of cellular division of hepatocytes was affected by the administration of adrenoblockers, oncogenic markers *α*-FP and c-Myc were evaluated. A markedly higher expression of both transcripts was detected in the cirrhosis and placebo groups compared to the hamsters treated with doxazosin (p<0.05), suggesting that this drug does not alter the normal process of cell growth. Carvedilol caused a slight rise in the expression of these two oncogenic markers in relation to the intact animals (Figure 5(b)).

The process of normal cell division in the liver was confirmed by assessing the level of HGF and CK-7 (Figures 5(a) and 5(b)). Whereas the concentration of HGF was similar in the doxazosin-treated and intact groups, it increased slightly but not significantly for the carvedilol and placebo groups. As aforementioned, the placebo group displayed a greater expression of HGF and c-Myc, evidencing possible abnormal cell growth. Regarding CK-7 (a marker of bile cell growth), the groups treated with doxazosin and carvedilol showed a 2-fold higher level compared to the intact group. With the doxazosin-treated hamsters, there were no significant differences in the concentration of c-Myc, HGF, or *α*-FP with respect to the intact animals, indicating that cell regeneration was probably adequate for the former group.

### 3.5. *α*/*β* Adrenoblockers (Doxazosin and Carvedilol) Reduce the Proliferation of Bile Type Cells (Ductular Response)

**Figure 6:**
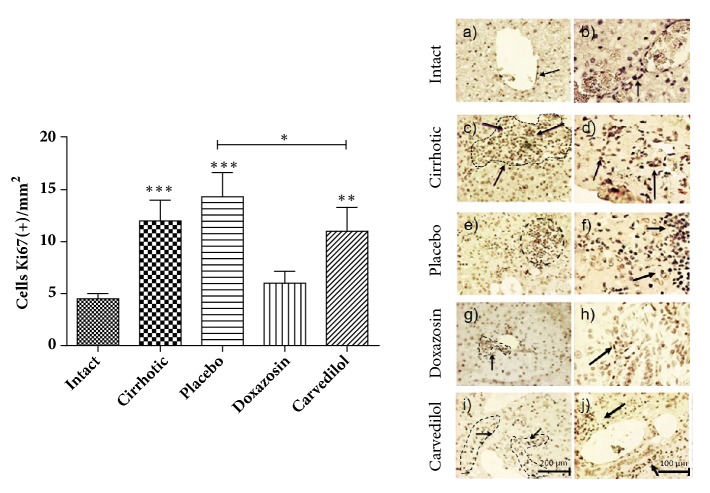
Immunohistochemistry for Ki-67 in the hepatic parenchyma to evaluate cell proliferation in the experimental animals treated with the alpha and beta adrenoblockers with respect to the intact, placebo, and cirrhotic controls. An increase in cell proliferation in the portal triad area (possibly a ductular response) is observed in the placebo and cirrhotic groups with respect to the treated and intact groups. Was evaluated with analysis of variance test with the Tukey post hoc. Values are expressed as the mean ± SD, ^*∗∗*^p < 0.01; ^*∗∗∗*^p < 0.001 of the groups carvedilol, cirrhosis, and placebo in relation to the intact; ^*∗*^p < 0.05 of the group carvedilol in relation to the placebo group (n=5).

To correlate proliferation changes, with the increase of markers c-Myc and *α*-FP, we analyzed an* in situ* Ki-positive cells in hepatic tissue from 50 mg/kg CCl4-induced hamsters treated with doxazosin, carvedilol, and the placebo group (control for endogenous reversion) (Figure 6). Positive Ki-67 cells (arrow) were observed in the area of the portal triad directly related to the bile duct generating a ductular response (dotted line, Figure 6). In the doxazosin group (Figures (g) and (h)) Ki-67 cells were 50% significantly lower than in the placebo group (Figures (e) and (f)) (^*∗∗*^p < 0.01). The carvedilol treated group (Figures (i) and (j)) showed an increase in the number of Ki-67 positive cells compared to the intact group (Figures (a) and (b)) (^*∗∗*^p < 0.01) and similar to the placebo group (Figures (e) and (f)). Taken together, these data indicated that doxazosin reduces the proliferation of possibly oval cells originating from the bile ducts.

## 4. Discussion

Various animal models have recently been employed for* in vivo* and* in vitro* experiments to investigate possible ways to revert liver cirrhosis [12, 17, 19]. To date, no conclusive findings have been reported that could establish the effectiveness of any given therapy. Due to the important participation of HSCs in hepatic fibrogenesis [12, 19, 20], they have been targeted in numerous studies seeking to diminish or revert fibrosis.

According to previous research by our group in a hamster model of CCI4-induced hepatic cirrhosis, treatment with doxazosin or carvedilol was found to reduce the concentration of collagen fibers in the cirrhotic liver and improve the functional restitution of the hepatic parenchyma by observing the decrease in ALT and AST and the increase in total protein synthesis [17] and, in addition, increases the presence of glycogen; as described in studies in rats with acetaminophen induced cirrhosis, when treated with HD-03 (plant extracts), rats improved their histology together with the liver function by increasing the glycogen reserve [3]. Another study conducted by Aldaba Muruato et al. (2013) demonstrated the recovery of liver glycogen in cirrhotic rats induced with CCl4 and treated with allopurinol [32]. Based on this background, it is demonstrated that the increase in glycogen synthesis capacity observed in our experiments is histological evidence that the effect of alpha and beta blockers recover liver function. Hamdy and El-Demerdash demonstrated that carvedilol has antifibrotic effects resulting from its antioxidant properties [12]. Since carvedilol treatment may contribute to the restauration of hepatic parenchyma and considering the *α*/*β* adrenoblocking function of these cells, the mechanism of action is possibly based on the inactivation of HSCs and a consequent reduction in collagen fibers. Nevertheless, the relation of doxazosin or carvedilol with the possible inactivation of HSCs has not yet been explored.

In the current study, the administration of each of the test drugs led to a lower concentration of *α*-SMA (a specific marker of HSCs) and TGF-*β* (a potent fibrogenic cytokine of HSCs). Hence, these adrenoblockers probably decrease the synthesis of both proteins by inhibiting the activation of HSCs [17, 19], leaving them in a quiescent or apoptotic state. The decline in the level of TGF-*β*, on the other hand, might have resulted from the effect of doxazosin on Kupffer cells (KCs), which represent one of the most important sources of production of this growth factor. Since the stimulation of *α*-1 adrenergic receptors of KCs activates these cells in a pathogenic manner by maintaining an inflammatory microenvironment, they are implicated in the activation of HSCs [33–35].

HSCs can regulate the expression of MMP-13, known to degrade type I and III collagen [36]. In the cirrhosis group, the level of MMP-13 decreased and that of TIMP-2 increased, thus inhibiting MMP activity [37]. Contrarily, the treatment with doxazosin or carvedilol increased the concentration of MMP-13 and decreased that of TIMP-2, which could explain the lower concentration of accumulated collagen in the parenchyma. The limited collagen found presently was similar to the amount caused by the administration of carvedilol in a model of fibrosis caused by the ligation of the bile duct, where the elevated expression of MMP-13 was accompanied by a reduced level of accumulated collagen [38].

In the placebo group, there was also a rise in the level of MMP-13 and a decline in the level of *α*-SMA. This is in accordance with a study on natural regeneration that found a reduction in fibrotic septa and a greater size of regenerative nodules during the endogenous regression of the process of cirrhosis [39].

We analyzed the effect of doxazosin and carvedilol in the process of cellular regeneration. Since c-Myc, *α*-FP, HGF, and CK-7 have been identified in events of cell proliferation, we used it as markers. c-Myc and *α*-FP are oncogene markers known to be highly expressed in various types of cancer, including hepatocellular carcinoma [40–43]. The levels of these markers were significantly increased in the placebo group in relation to the intact group where it presents a low expression of these proliferation markers (plots of amplification in supplementary materials (available here)), which explains the larger size of regenerative nodules detected with histological techniques [39–41, 44]. Further research is needed to clarify the significant rise in the concentration of c-Myc in the group treated with carvedilol.

No significant difference existed in the expression of the HGF gene between any of the groups, although in literature this gene is reportedly implicated in normal cellular regeneration and possibly was elevated in the groups treated during a time prior to the analysis. It was previously found to be highly elevated in partially hepatectomized rats, thus favoring cellular regeneration in the liver [45]. Lastly, an increased level of CK-7 was observed in the animals treated with either of the two *α*/*β* adrenoblockers, thus demonstrating an enhanced proliferation of cells in bile ducts [46].

Ki-67 protein is expressed during the G1, G2, and S phases of cell division. In this study, the number of Ki-67-positive cells in the cirrhotic group by CCl4 and placebo were higher than in the group treated with doxazosin. This proliferation marker was identified in cells near the bile ducts of the portal triad, suggesting an increase in immature liver cells, known as ductular response, correlating this increase in cell proliferation with the increase of c-Myc [47–49]

## 5. Conclusion

In hamsters with CCI4-induced hepatic cirrhosis, both the doxazosin and carvedilol treatment gave rise to an improvement in the liver function and the architecture with the reduction of regeneration nodules due to fibrosis generated. However, we observed that doxazosin caused slight morphological changes in hepatocytes, such as its balonization but did not alter the normal processes of regeneration of hepatic parenchyma with respect to a possible increase in proliferation markers or loss of function. On the other hand in animals treated with carvedilol, it elicited a slight irregular expression of cell proliferation markers such as c-Myc and Ki-67.

## Figures and Tables

**Table 1:** Oligonucleotides used for qPCR.

**Gene**	**Oligonucleotide-F**	**Oligonucleotide-R**	**Accession Number**
**CK-7**	GACCCTACCATCCAGCAAGT	CCACTTGGTCTCCAGCATCT	XM_005067271.2
***α*-FP **	GAGATTGAGAAGCTGGTCCTG	GCAGCACTCTGCTATTTTGG	XM_005068161.1
**c-Myc**	TGCTCCACCTCTAGCCTGTA	AGGAGAGAAGGCTGTGGAGT	AJ582076.1
**HGF**	GCATCATTGGTAAAGGAGGC	GCGTACCTCTGGATTGCTTG	XM_005080880.1
**TIMP-2**	TCAAAGGCCCTGACAAAGAC	AGGCTCTTCTTCTGGGTGGT	XM_007634128.1
**MMP-13**	TGTCCTGGCCACTCTCTTCT	GGGTCATCAAGTTTGCCAGT	XM_005077319.1
**Beta-actin**	GCCCAGAGCAAGAGAGGTAT	CACGCAGCTCGTTGTAGAAG	XM_013120404.1

## Data Availability

The data used to support the findings of this study are available from the corresponding author upon request.

## Supplementary Materials

Supplementary Materials Two images of the amplification plots are presented with the replicas obtained for alpha-fetoprotein by qPCR for the different experimental animals treated and cirrhotic and intact controls. Where the amplification cycles are observed, the alpha-fetoprotein begins to be detected according to the threshold.
