# Supplementary figures and images for: Doxazosin and Carvedilol Treatment Improves Hepatic Regeneration in a Hamster Model of Cirrhosis
Source: Biomed Res Int. 2018 Dec 12;2018:4706976. doi: 10.1155/2018/4706976 (PMC6311259; doi:10.1155/2018/4706976)

## Slide 1
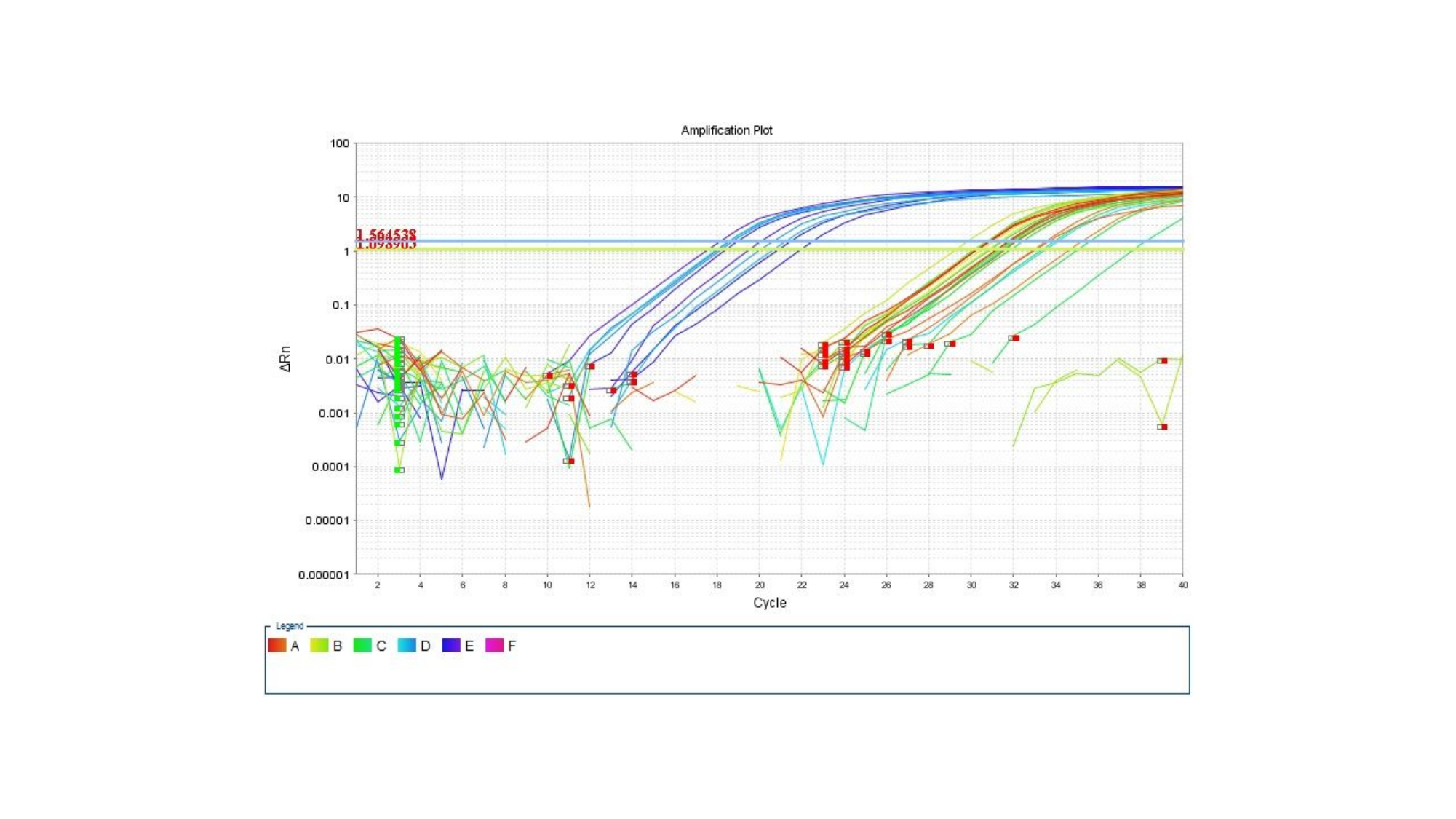

## Slide 2
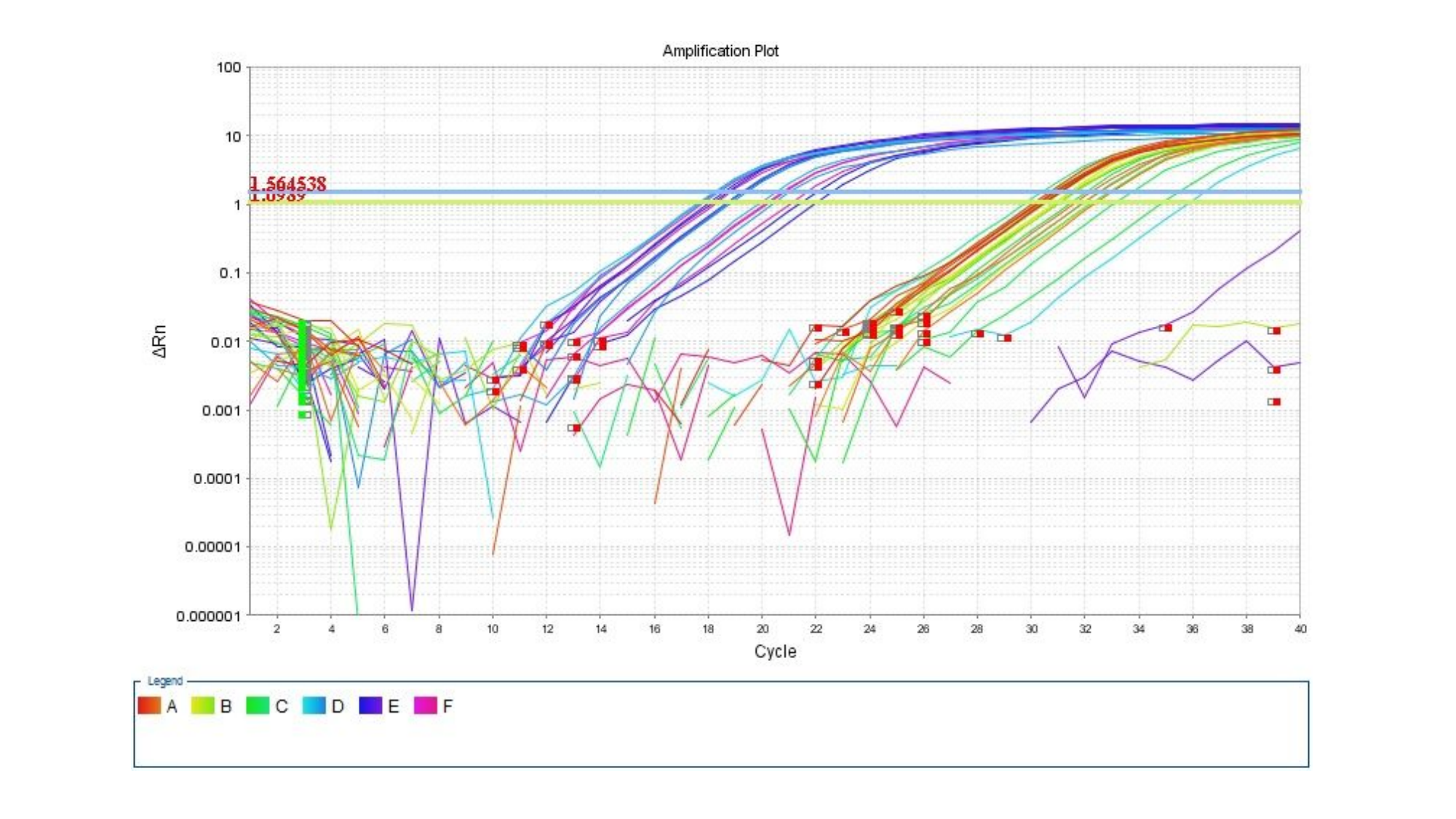

Supplement: Supplementary Materials — Two images of the amplification plots are presented with the replicas obtained for alpha-fetoprotein by qPCR for the different experimental animals treated and cirrhotic and intact controls. Where the amplification cycles are observed, the alpha-fetoprotein begins to be detected according to the threshold. [file 4706976.f1.pptx]
